# L´intérêt de la piézochirurgie dans le traitement d’une dysplasie cémento-osseuse surinfectée par actinomycose: à propos d'un cas

**DOI:** 10.11604/pamj.2021.38.106.27167

**Published:** 2021-02-02

**Authors:** Imen Cherni, Hajer Hentati, Rim Hadhri, Adel Bouguezzi, Abdellatif Chokri, Jamil Selmi

**Affiliations:** 1 Service de Médecine et Chirurgie Buccales, Clinique Universitaire de Médecine et Chirurgie Dentaire, Monastir, Tunisie,; 2 Laboratoire de Recherche Santé Orale et Réhabilitation Bucco-Faciale LR12ES11, Faculté de Médecine Dentaire de Monastir, Université de Monastir, Monastir, Tunisie,; 3 Service d'Anatomie et Cytologie Pathologiques, Hôpital Fattouma Bourguiba, Monastir, Tunisie

**Keywords:** Dysplasie cémento-osseuse, actinomycose, piézochirurgie, à propos d’un cas, Cemento-osseous dysplasia, actinomycosis, piezosurgery, case report

## Abstract

La dysplasie cémento-osseuse est une lésion fibro-osseuse non néoplasique qui touche l´os alvéolaire. Elle peut se présenter sous l´une des trois formes: périapicale, focale ou floride. Elle est souvent asymptomatique de découverte fortuite lors d´un examen radiologique de routine. Cependant, elle peut devenir symptomatique suite à une surinfection, une fois exposée à la flore bactérienne buccale. Nous rapportons un cas de dysplasie cémento-osseuse floride associée à une actinomycose osseuse chez une femme tunisienne de 53ans. Cette surinfection est rarement évoquée dans la littérature; une recherche sur PubMed utilisant la formule booléenne « cemento-osseous dysplasia AND actinomyces » a révélé un seul article (Smith et al. 2011). Le traitement de l´infection actinomycosique nécessite souvent une antibiothérapie de longue durée parfois associée à une chirurgie de débridement comme dans ce cas où la piézochirurgie a été utilisée dans l´élimination de la dysplasie et de l´os nécrotique.

## Abstract (en)

Cemento-osseous dysplasia is a benign fibro-osseous lesion affecting the alveolar bone. It is classified into three forms: periapical, focal or florid dysplasia. It is often asymptomatic and fortuitously discovered during a routine radiological examination. However, it may become symptomatic after superinfection, after patient's exposure to oral bacterial flora. We here report a case of florid cemento-osseous dysplasia associated with actinomycosis of bone in a 53-year-old Tunisian woman. This superinfection has been rarely reported in the literature; Boolean Searching on PubMed for the keywords “cemento-osseous dysplasia AND actinomyces” displays a single article (Smith et al. 2011). The treatment of actinomycosis infection often requires long-term antibiotic therapy, sometimes associated with surgical debridement, as in the case of this patient who underwent piezosurgery to treat dysplasia and necrotic bone.

## Introduction

Les actinomycètes sont des bactéries anaérobies gram positif qui font partie de la flore commensale surtout de la cavité buccale et de l´oropharynx. La rupture des barrières anatomiques constituées par les muqueuses et la peau peut être la cause d´une infection rare mais grave dite actinomycose dont la localisation cervico-faciale est la plus fréquente. La propagation de l´infection se fait généralement par contigüité mais la dissémination par voie hématique et lymphatique vers d´autres organes reste possible [1]. L´actinomycose est plus observée au niveau des tissus mous qu´au niveau osseux. La localisation osseuse est due souvent à l´extension de l´infection superficielle et rarement suite à un traumatisme [2]. La dysplasie cémento-osseuse est caractérisée par une architecture osseuse perturbée, et remplacée par un stroma fibreux avec une phase minérale importante (os, ostéoïde, cément.) et une faible vascularisation qui peut être la cause de nécrose et de séquestration si une infection se développe au sein de cet os dysplasique [2,3]. A travers ce cas clinique on va discuter le diagnostic clinique, radiologique et histopathologique de ces deux pathologies ainsi que l´attitude thérapeutique face à leur association.

## Patient et observation

**Figure 1:**
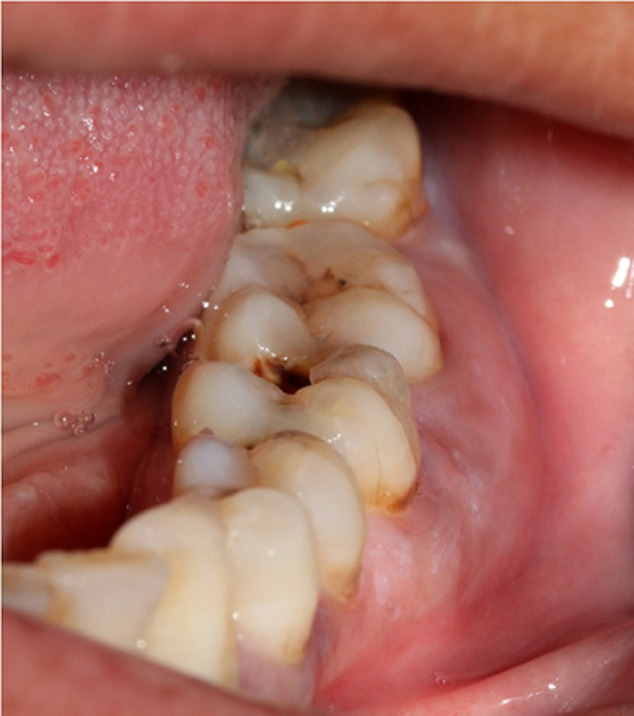
comblement du fond du vestibule mandibulaire gauche en regard de la 36 cariée

**Figure 2:**
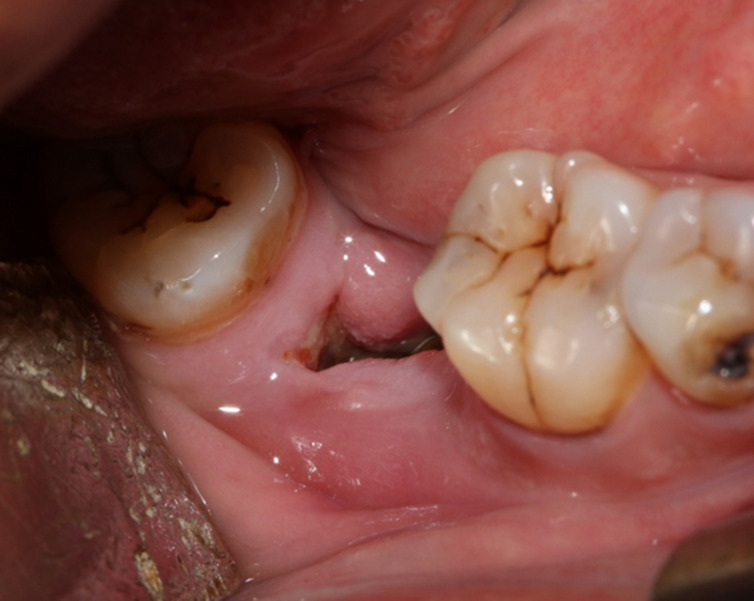
ulcération à l´emplacement de la 47 exposant un os jaunâtre

**Figure 3:**
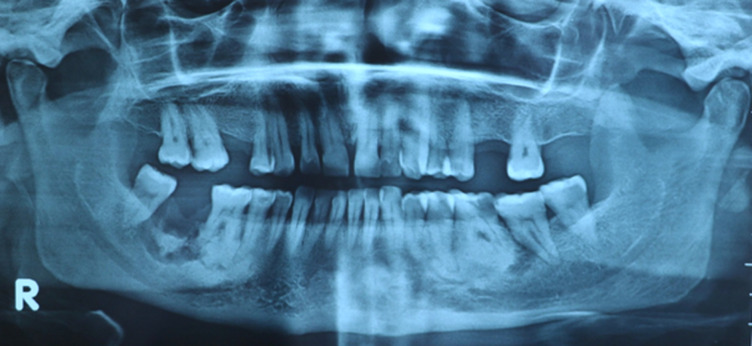
radiographie panoramique montrant des radio-opacités multiples

**Figure 4:**
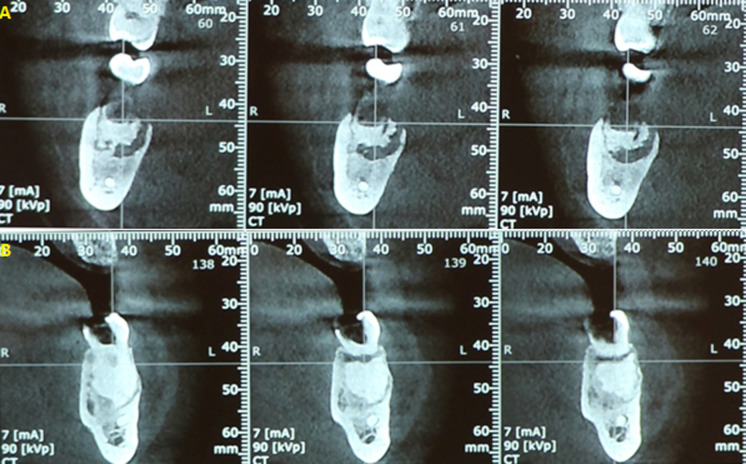
Cone Beam mandibulaire; A) calcification appendue à l´apex de la 36 cariée; B) calcification au niveau du site de la 47 extraite

Une patiente âgée de 53 ans, en bon état de santé général, a été adressée, par un confrère, au service de médecine et chirurgie buccales de la clinique universitaire de médecine et chirurgie dentaire de Monastir pour une tuméfaction génienne basse gauche. La patiente a été déjà mise sous antibiotique Augmentin®1g*2/jour depuis deux jours. L´examen exobuccal a retrouvé la tuméfaction. L´examen endobuccal a montré un comblement vestibulaire en regard de la 36 cariée (Figure 1); l´hygiène bucco-dentaire était défectueuse avec des dépôts de plaque et de tartre. Le reste de l´examen dentaire a montré des dents cariées (la 17, la 24, la 38 et la 45) et des dents absentes suite à leur extraction (la 15, la 26, la 28 et la 47). A l´emplacement de la 47, une ulcération muqueuse d´environ 1,5 cm de grand axe exposant un os jaunâtre, non douloureux a été révélée (Figure 2). La patiente a rapporté qu´elle ne s´est pas rendu compte de cette ulcération et que la 47 a été extraite 14 ans auparavant sans suites notables. La radiographie panoramique a montré des images radio-opaques mandibulaires multiples: du côté gauche, elles sont appendues aux apex de la 35 et de la 36; antérieurement elles sont localisées au niveau de la région péri-apicale de la 33; du côté droit elles sont appendues aux apex de la 45 et de la 46 et aussi au niveau du site d´extraction de la 47 où la radio-opacité était entourée d´un halo radioclaire assez large (Figure 3). Une tomographie volumique à faisceau conique (Cone Beam) a été demandée pour complément d´informations; elle a retrouvé les lésions sus-décrites. (Figure 4 A, B)

**Figure 5:**
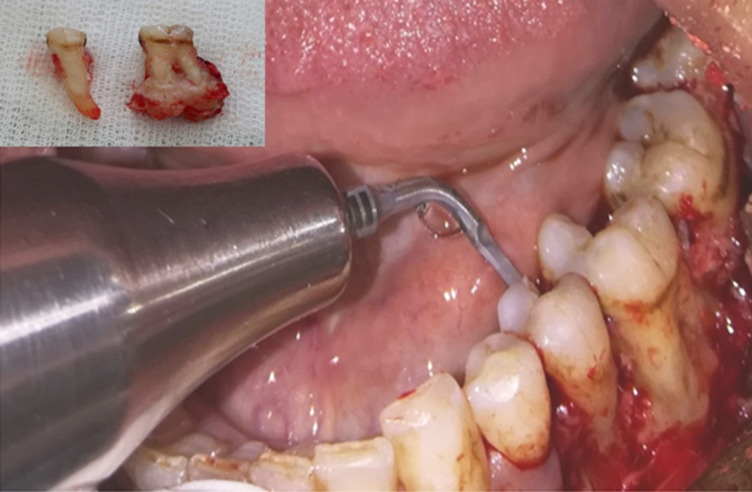
piézochirurgie pour élimination des calcifications apicales et extraction de la 35 et la 36

**Figure 6:**
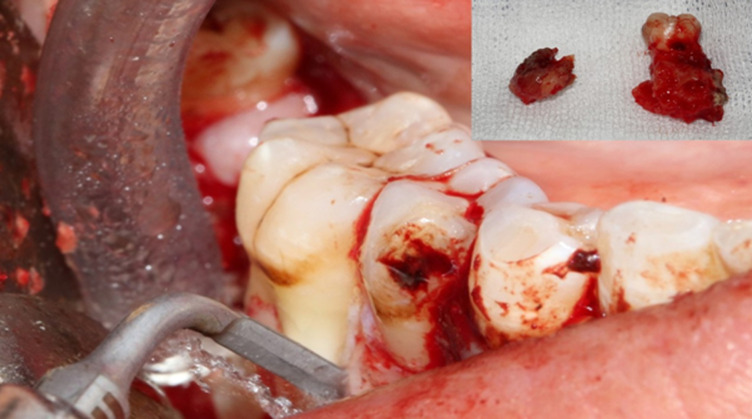
piézochirurgie pour élimination de la calcification au niveau de l´emplacement de la 47 extraite et extraction de la 46

**Figure 7:**
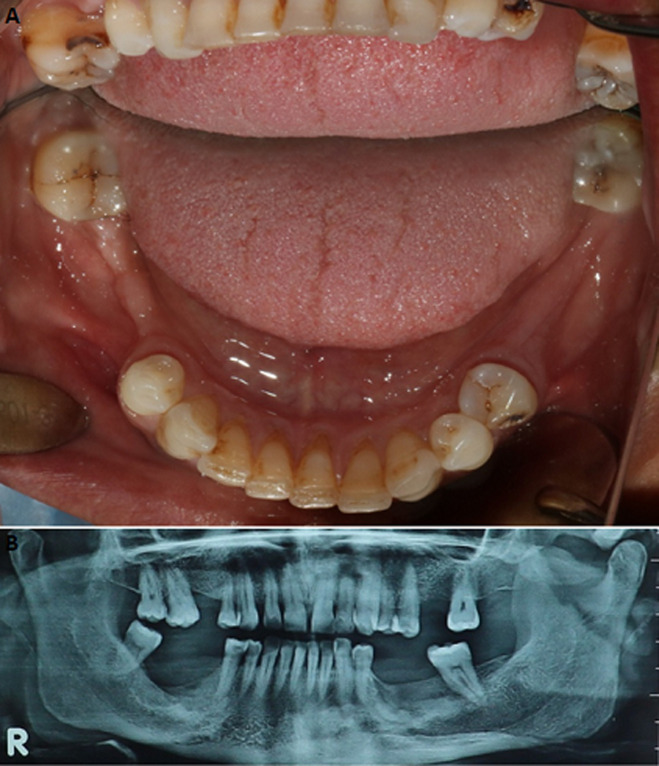
contrôle clinique et radiologique 6 mois après la chirurgie; A) contrôle clinique après 6 mois; B) contrôle radiologique après 6 mois

Devant les données de l´examen clinique et radiologique, le diagnostic d´une dysplasie cémento-osseuse mandibulaire floride surinfectée a été évoqué. Le traitement chirurgical a été réalisé en deux temps: du côté gauche, on a procédé à l´extraction de la 35 et la 36 avec élimination des calcifications apicales en utilisant la technique piézochirurgicale (Figure 5). L´examen histopathologique a confirmé le diagnostic de dysplasie cémento-osseuse surinfectée. La 38 cariée a été extraite ultérieurement. Du côté droit, la piézochirurgie a été réutilisée pour faire l´exérèse de l´os exposé ainsi que l´extraction de la 46 avec le tissu dysplasique apical (Figure 6). Un curetage de la cavité résiduelle a été effectué. L´examen histopathologique a mis en évidence des travées osseuses délimitant des espaces fibreux comportant un infiltrat inflammatoire abondant fait de lymphocytes, plasmocytes et polynucléaires neutrophiles. Au sein de cet infiltrat, des grains d’actinomycose ont été identifiés. Le diagnostic était en faveur d´une dysplasie cémento-osseuse surinfectée associée à une actinomycose. En post-chirurgical, la patiente a été mise sous antibiotiques Augmentin® (amoxcilline + acide clavulanique), paracétamol et chlorhexidine durant 10 jours. L´antibiothérapie, à base d´amoxicilline seule a été prolongée pendant 3 mois avec des contrôles cliniques et radiologiques réguliers avec un recul actuel de 12 mois (Figure 7 A, B).

## Discussion

L´actinomycose est une infection granulomateuse, chronique, rare mais grave, causée par une bactérie anaérobie ou aérobie facultative du genre *actinomycès* qui fait partie de la flore commensale gastro-intestinale et génitale [2,4]. En cas de perte de l´intégrité des muqueuses, la bactérie peut devenir pathogène en envahissant les tissus sous-jacents. L´actinomycose peut toucher plusieurs organes. Les formes les plus répandues par ordre décroissant sont: l´actinomycose cervico-faciale, l´actinomycose abdominale, l´actinomycose thoracique et pelvienne. *L´actinomycès israelii* est le plus incriminé dans l´actinomycose humaine [4]. L´actinomycose cervico-faciale est souvent indolore et peut se présenter sous forme d´ulcération, tuméfaction, abcès avec parfois des fistules qui drainent des grains jaunâtres. Elle touche souvent les tissus mous mais rarement l´os sous-jacent. Elle est favorisée par la mauvaise hygiène [1]. L´examen histopathologique permet généralement de poser le diagnostic d´actinomycose par la mise en évidence des grains actinomycosiques comme c´est le cas; ce sont des granules de moins de 1mm de diamètre ayant une structure filamenteuse qui ressemble à une touffe de cheveux formées par des colonies d´actinomycètes. Ces granules peuvent être observées également au niveau des infections par le *Nocardia brasiliensis* et par le *Streptomyces madurae* [1,4].

L´examen bactériologique demeure l´examen de certitude permettant l´identification exacte de l´espèce causal. Cependant cet examen est un peu délicat nécessitant une mise en culture dans des conditions d´anaérobiose stricte avec un transport rapide vers le laboratoire (moins de 15 minutes) dans un milieu anaérobie [4]. Ces germes sont sensibles aux beta-lactamines, tetracyclines, macrolides, clindamycine, carbapénème, alors que le métronidazole, les aminoglycosides, l´aztréonam, le cotrimoxazole, l´oxacilline, la céfalexine, et les fluoroquinolones n´ont aucun effet sur *l´actinomycès* [2]. Le traitement conventionnel de l´actinomycose était basé sur une antibiothérapie massive, de longue durée commençant par l´administration de la pénicilline G en intra-veineux avec des doses élevées de 18 à 24 MU durant 2 à 6 semaines, puis un relais par la pénicilline V ou l´amoxicilline pendant 6 à 12 mois [4]. Moghini *et al*. ont prouvé l´efficacité d´une antibiothérapie de courte durée (moins de 2 mois) associée souvent à un traitement chirurgical pour la guérison de certaines formes d´actinomycose notamment l´actinomycose cervico-faciale [5]. L´intervention chirurgicale par débridement et curetage de la lésion pourrait expliquer la bonne réponse au traitement médical de courte durée; elle permet d´améliorer l´action des antibiotiques et favorise leur diffusion aux germes *d´actinomycès*. Ces germes étaient sous une forme de résistance rassemblés en grains actinomycosiques entourés de fibrose tissulaire avec faible vascularisation et par la suite faible diffusion d´antibiotique [5,6].

La durée de traitement médical est donc à ajuster selon l´évolution clinique et radiologique d´où la nécessité des rendez-vous de contrôle réguliers [7]. Dans notre cas l´actinomycose s´est développée sur une dysplasie cémento-osseuse préexistante suite à une extraction dentaire qui l´a exposée au niveau de la cavité buccale. La dysplasie cémento-osseuse est une lésion fibro-osseuse non néoplasique qui touche l´os alvéolaire, dite aussi dysplasie osseuse ou dysplasie cémentaire ou céméntome. Elle se voit surtout chez les femmes adultes de race noire dont on décrit trois formes: la dysplasie cémento-osseuse périapicale à localisation antéro-inférieure, la dysplasie focale localisée au niveau d´une seule dent et la dysplasie floride qui est multifocale comme dans ce cas clinique [3]. Sur le plan radiologique, ces lésions peuvent se présenter sous forme d´une image radioclaire au début de leur formation, image mixte, radio-opaque entourée d´un halo radioclair ou image radio-opaque mature [3,8]. Sur le plan histopathologique, on décrit une perturbation de la structure lamellaire osseuse, remplacée par un stroma fibreux avec des dépôts des tissus minéralisés ressemblant au cément. Plus les lésions sont matures, plus la calcification est importante et la vascularisation est faible ce qui fait que l´os dysplasique est moins résistant à l´infection qui peut se produire suite à une nécrose pulpaire, une maladie parodontale, une irritation due au port des prothèses non stables ou une extraction dentaire qui expose la dysplasie au niveau de la cavité buccale comme le cas de notre patiente [3,9].

Face à une dysplasie cémento-osseuse surinfectée, le traitement chirurgical s´impose par résection et curetage. L´antibiothérapie seule n´est pas suffisante vue le caractère avasculaire de ces lésions [9]. La piézochirurgie peut faciliter l´abord chirurgical de ces lésions; cette technique a été utilisée pour la première fois par Vercelotti en 1988 à visée implantaire puis elle a été introduite en chirurgie orale mais également dans d´autres domaines comme la neurochirurgie, l´ophtalmologie, l´otorhinolaryngologie, l´orthopédie et la traumatologie [10]. Cette technique est basée sur l´effet piézoélectrique qui consiste à la déformation de certains cristaux et céramiques lorsqu´ils sont placés dans un champ électrique donnant naissance à des ondes ultrasoniques qui sont amplifiées et transférées à une pointe vibrante ayant un effet de coupe sur les tissus minéralisés [10]. L´appareil de piézochirurgie utilisé (PIEZOSURGERY 2, SATELEC, ACTEON EQUIPMENT) est formé d´une pièce à main connectée à l´unité principale et de différents types et formes d´inserts dont chacun s´adapte à une situation clinique bien définie. Dans notre cas on a eu recours à deux kits; le kit de l´avulsion dentaire par l´insert LC1 afin d´effectuer une syndesmotomie profonde et celui de la chirurgie osseuse par l´insert BS6 ayant la forme d´un scalpel incurvé qui nous a aidé à séparer entre deux surfaces : l´os sain et l´os dysplasique. La piézochirurgie offre une précision de coupe sans endommager les dents adjacentes et les structures anatomiques nobles de voisinage. Elle permet aussi grâce au système d´irrigation intégré, d´éviter l´échauffement osseux induit par les instruments rotatifs conventionnels qui pourrait engendrer une nécrose osseuse iatrogène [10].

## Conclusion

Pour conclure, si une dysplasie cémento-osseuse est découverte de façon fortuite lors d´un examen radiologique de routine, l´extraction doit être évitée au maximum et le patient doit être suivi régulièrement pour détartrage et soin de carie afin d´éviter une éventuelle surinfection particulièrement par l´actinomycose qui nécessite à la fois un traitement médical et chirurgical.

## References

[1] Badre B, Essaadi M, El Arabi S (2013). L´actinomycose cervico-faciale: à propos d'un cas. Pan Afr Med J.

[2] Smith MH, Harms PW, Newton DW, Lebar B, Edwards SP, Aronoff DM (2011). Mandibular actinomyces osteomyelitis complicating florid cemento-osseous dysplasia: case report. BMC Oral Health.

[3] El-Naggar AK, Chan JK, Grandis JR, Takata T, Slootweg PJ (2017). WHO classification of head and neck tumours. Lyon. International agency of research on cancer.

[4] Boyanova L, Kolarov R, Mateva L, Markovska R, Mitov I (2015). Actinomycosis: a frequently forgotten disease. Future Microbiol.

[5] Moghimi M, Salentijn E, Debets-Ossenkop Y, Karagozoglu KH, Forouzanfar T (2013). Treatment of cervicofacial actinomycosis: a report of 19 cases and review of literature. Med Oral Patol Oral Cir Bucal.

[6] Nagler R, Peled M, Laufer D (1997). Cervicofacial actinomycosis: a diagnostic challenge. Oral Surg Oral Med Oral Pathol Oral RadiolEndod.

[7] Sudhakar SS, Ross JJ (2004). Short-term treatment of actinomycosis: two cases and a review. Clin Infect Dis.

[8] Massereau E, Ordioni U, Guivarc´h M, Royer G, Catherine JH (2015). Dysplasie osseuse floride mandibulaire: un cas de découverte fortuite et revue de la littérature. Med Buccale Chir Buccale.

[9] Kato CNAO, de Arruda JAA, Mendes PA, Neiva IM, Abreu LG, Moreno A (2020). Infected cemento-osseous dysplasia: analysis of 66 cases and literature review. Head Neck Pathol.

[10] Pavlíková G, Foltán R, Horká M, Hanzelka T, Borunská H, SedÃ½ J (2011). Piezosurgery in oral and maxillofacial surgery. Int J Oral MaxillofacSurg.

